# Beetroot (*Beta vulgaris* L.) Extract Ameliorates Gentamicin-Induced Nephrotoxicity Associated Oxidative Stress, Inflammation, and Apoptosis in Rodent Model

**DOI:** 10.1155/2014/983952

**Published:** 2014-10-22

**Authors:** Ali A. El Gamal, Mansour S. AlSaid, Mohammad Raish, Mohammed Al-Sohaibani, Shaza M. Al-Massarani, Ajaz Ahmad, Mohamed Hefnawy, Mohammed Al-Yahya, Omer A. Basoudan, Syed Rafatullah

**Affiliations:** ^1^Department of Pharmacognosy and Medicinal, Aromatic & Poisonous Plants Research Center (MAPPRC), College of Pharmacy, P.O. Box 2457, King Saud University, Riyadh 11451, Saudi Arabia; ^2^Department of Pharmacognosy, College of Pharmacy, Mansoura University, El Mansoura 35516, Egypt; ^3^Department of Pharmaceutics, College of Pharmacy, P.O. Box 2457, King Saud University, Riyadh 11451, Saudi Arabia; ^4^Department of Medicine and Pathology, Gastroenterology Unit, Collage of Medicine, King Khalid University Hospital, King Saud University P.O., Box 2925, Riyadh 11461, Saudi Arabia; ^5^Department of Clinical Pharmacy, College of Pharmacy, P.O. Box 2457, King Saud University, Riyadh 11451, Saudi Arabia; ^6^Department of Pharmaceutical Chemistry, College of Pharmacy, P.O. Box 2457, King Saud University, Riyadh 11451, Saudi Arabia

## Abstract

The present investigation was designed to investigate the protective effect of (*Beta vulgaris* L.) beat root ethanolic extract (BVEE) on gentamicin-induced nephrotoxicity and to elucidate the potential mechanism. Serum specific kidney function parameters (urea, uric acid, total protein, creatinine, and histopathology of kidney tissue) were evaluated to access gentamicin-induced nephrotoxicity. The oxidative/nitrosative stress (Lipid peroxidation, MDA, NP-SH, Catalase, and nitric oxide levels) was assessed. The inflammatory response (TNF-*α*, IL-6, MPO, NF-*κ*B (p65), and NF-*κ*B (p65) DNA binding) and apoptotic marker (Caspase-3, Bax, and Bcl-2) were also evaluated. BVEE (250 and 500 mg/kg) treatment along with gentamicin restored/increased the renal endogenous antioxidant status. Gentamicin-induced increased renal inflammatory cytokines (TNF-*α* and IL-6), nuclear protein expression of NF-*κ*B (p65), NF-*κ*B-DNA binding activity, myeloperoxidase (MPO) activity, and nitric oxide level were significantly down regulated upon BVEE treatment. In addition, BVEE treatment significantly reduced the amount of cleaved caspase 3 and Bax, protein expression and increased the Bcl-2 protein expression. BVEE treatment also ameliorated the extent of histologic injury and reduced inflammatory infiltration in renal tubules. These findings suggest that BVEE treatment attenuates renal dysfunction and structural damage through the reduction of oxidative stress, inflammation, and apoptosis in the kidney.

## 1. Introduction

The beetroot (*Beta vulgaris* L.), locally known as Shamandar, is a vegetable plant and belongs to family Amaranthaceae. The roots of beet have long been used in traditional Arab medicine to treat a wide variety of diseases. The claimed therapeutic use of beetroot includes its antitumor, carminative, emmenagogue, and hemostatic and renal protective properties and is a potential herb used in cardiovascular conditions [1]. Beetroot is known to be a powerful antioxidant [2]. In ancient times, beetroot was believed to help enhance human sex hormones and as an aphrodisiac. The juice of beetroot is also consumed as a natural remedy for sexual weakness and to expel kidney and bladder stones [3]. In recent years, beetroot has gained popularity to be a natural food to boost the energy in athletes [4, 5]. The beetroot leaves were recommended by the Father of Medicine “Hippocrates” for faster healing of wounds [6]. Recent reports indicate that* Beta vulgaris* extracts (root) possess antihypertensive, hypoglycemic, antioxidant [7], anti-inflammatory, and hepatoprotective activities [6, 8–10]. Previously, red beetroot extract has been demonstrated to be an effective multiorgan tumor suppressing agent in laboratory animals [9, 11, 12].

Gentamicin (GM), an aminoglycoside, is known for its nephrotoxicity and one of the possible mechanisms suggested is damage due to generation of free radicals. [13, 14]. GM induces a dose-dependent nephrotoxicity in 10–25% of therapeutic courses [15]. There are reports which suggest the role of ROS/Nitrogen species, in association with increased lipid peroxide formation and decreased activity of antioxidant enzymes in GM-induced nephrotoxicity [16]. Recent studies have also postulated that renal inflammation, which is characterized by infiltration of inflammatory cells such as monocytes/macrophages and subsequent release of proinflammatory cytokines and activation of NF-*κ*B in response to oxidative stress, is involved in this process [17, 18]. Furthermore, induced apoptosis/necrosis of renal tubular epithelial cells [19–21].

From above literature,* Beta vulgaris* is known to possess potent antioxidant, anti-inflammatory properties. Therefore, the present study was carried out to evaluate the renoprotective effect of* Beta vulgaris *ethanol extract (BVEE) against gentamicin induced nephrotoxicity in rats to substantiate its use in Arab traditional medicine.

## 2. Materials and Methods

### 2.1. Drugs and Chemicals

GM sulfate was obtained from Abbott Healthcare PVT Ltd, India. 2-thiobarbituric acid (TBA), antibodies against NF-*κ*B (p65), cleaved caspase-3, Bax, Bcl-2, *β*-actin, and HRP-conjugated secondary antibody were purchased from Santa Cruz biotech (USA). NE-PER nuclear and cytoplasmic extraction kit was obtained from Pierce Biotechnology, Rockford, IL, (USA). NF-*κ*B (p65) transcription factor assay kit was obtained from Cayman Chemical Company, Ann Arbor, MI. Rat TNF-*α* and IL-6 ELISA kits were obtained from R&D Systems, Inc. (USA). All other chemicals were of analytical grade.

### 2.2. Plant Material

The fresh beetroots were obtained from a local market in Riyadh. They were identified and authenticated by taxonomist Professor Mohammed Youssef, Department of Pharmacognosy, College of Pharmacy, King Saud University. A voucher specimen (number 0214) was deposited at the Herbarium of the College of Pharmacy, King Saud University, Riyadh, Saudi Arabia.

### 2.3. Preparation of the Extract and Fractions

The fresh roots of* Beta vulgaris* (1 kg, cut into small pieces) were exhaustively macerated by soaking in 70% (1.5 L) ethanol and the process repeated for three successive days. The obtained alcoholic extract was then concentrated under reduced pressure using rotatory evaporator till complete drying. The resulted extract (BVEE, 150 g) was later suspended in distilled water and evaluated for nephroprotective activity.

### 2.4. Scavenging Ability towards 2, 2-Diphenyl-1-picrylhydrazyl (DPPH) Radicals

The method was carried out as described by Brand-Williams et al., 1995 [22]. Various concentrations (10, 50, 100, 500, and 1000 *μ*g/mL) of the crude extract and fractions were used. The assay mixtures contained in total volume of 1 mL, 500 *μ*L of the extract, 125 *μ*L prepared DPPH, and 375 *μ*L solvent. Ascorbic acid was used as the positive control. After 30 min incubation at 25°C, the decrease in absorbance was measured at *λ* = 517 nm. The radical scavenging activity was calculated from the following:

(1) %radical  scavenging  activity=((Acontrol−Asample)Acontrol)×100,

where *A*_sample_ and *A*_control_ are absorbance of sample and control, respectively. The decrease in DPPH solution absorbance indicates an increase of DPPH radical scavenging activity of extract tested. The antioxidant activity was expressed as the number of equivalents of ascorbic acid.

### 2.5. Animals

Wistar albino rats, of either sex and approximately of same age (8–10 weeks), weighing 180–200 g was obtained from the Animal Care Center, College of Pharmacy, King Saud University, Riyadh, Saudi Arabia. Animals were kept at a constant temperature (22 ± 2°C), humidity (55%), and light-dark conditions (12/12 h light/dark ratio). Animals were provided with Purina chow diet and drinking water* ad-libitum*. The protocol of the current study was approved by the Ethics Committee of the Experimental Animal Care Society, College of Pharmacy, King Saud University, Riyadh, Saudi Arabia.

### 2.6. Acute Toxicity Test

The acute toxicity test was performed on rats using oral route. Beet root extract was dissolved in distilled water and administered at various doses, ranging from 50 to 2000 mg/kg to the groups of rats. The animals were observed continuously for 1 h and then at half-hourly intervals for 4 h on the first day, for clinical signs and symptoms of toxicity further up to 72 h followed by 14 days for any mortality [23].

### 2.7. Experimental Design

Animals were divided into four groups (I, II, III, and IV) (*n* = 6 animals/group). Group I was kept as a control (no treatment). Groups II, II, and IV received gentamicin. GM was administered to group 2 in doses of 85 mg/kg body weight intraperitoneally (i.p.) daily for 8 days [24]. Groups 3 and 4 were treated with BVEE at the doses of 250 and 500 mg/kg weight (orally), respectively, for 20 days before GM treatment and there after concurrently with GM (85 mg/kg) for 8 days. The blood samples were collected after 24 h of last dose. The blood was collected and the serum was separated for biochemical estimations. After blood collection, the animals were sacrificed using ether anesthesia. The kidneys were dissected out and used for biochemical, molecular, and histopathological studies.

### 2.8. Serum Analysis

Creatinine [25], uric acid [26], and urea [27] levels were estimated in serum using Reflotron Plus Analyzer and Roche Kits (Roche Diagnostics).

### 2.9. Determination of Malondialdehyde (MDA)

The method reported by Utley et al., 1967 [28], was followed. The kidney tissues were removed and each tissue was homogenized in 0.15 M KCl (at 4°C; Potter-Eivehjem type C homogenizer) to give a 10% w/v homogenate. Aliquots of homogenate (1 mL) were incubated at 37°C for 3 h in a metabolic shaker. Then 1 mL of 10% aqueous trichloroacetic acid was added and mixed. The mixture was then centrifuged at 800 g for 10 min. 1 mL of the supernatant was removed and mixed with 1 mL of 0.67% thiobarbituric acid in water and placed in a boiling water bath for 10 min. The mixture was cooled and diluted with 1 mL distilled water. The absorbance of the solution was then read at 535 nm. The content of malondialdehyde (nM/g wet tissue) was then calculated, by reference to a standard curve of malondialdehyde solution.

### 2.10. Estimation of Nonprotein Sulfhydryl's (NP-SH) Content in Kidney Tissue

Renal nonprotein sulfhydryl's was measured according to the method of Sedlak and Lindsay, 1968 [29]. The kidney was homogenized in ice-cold 0.02 mM/L ethylenediaminetetraacetic acid (EDTA). Aliquots of 5 mL of the homogenates were mixed in 15 mL test tubes with 4 mL of distilled water and 1 mL of 50% trichloroacetic acid (TCA). The tubes were shaken intermittently for 10 min and centrifuged at 3000 rpm for 10 min. Two milliliters of supernatant was mixed with 4 mL Tris buffer (0.4 mol/L) (pH 8.9). 0.1 mL of 5,5′-dithiobis(2-nitrobenzoic acid) (DTNB) was added and the sample was shaken. The absorbance was measured within 5 min of addition of DTNB at 412 nm against a reagent blank. The content of NPSH was calculated using spectrophotometer.

### 2.11. Estimation of Catalase and Total Protein Content of Kidney Tissue

The catalase was estimated by the kit method, supplied by Crescent Diagnostics, Jeddah, Saudi Arabia.

### 2.12. Myeloperoxidase (MPO) Level of Kidney Tissue

MPO assay neutrophil recruitment was indirectly measured by means of MPO activity, determined according to the method described previously by Krueger et al. 1990 [30]. Samples were homogenized at 5% (w/v) in EDTA/NaCl buffer (pH 4.7) and centrifuged at 10,000 rpm for 15 min at 4°C. The pellet was resuspended in 0.5% hexadecyl-trimethyl ammonium bromide buffer (pH 5.4), and the samples were frozen and thawed three times in liquid nitrogen. Upon thawing, the samples were recentrifuged (10,000 rpm, 15 min, 4°C), and 25 *μ*L of the supernatant was used for the MPO assay. The enzymatic reaction was assessed with 1.6 mM tetramethylbenzidine, 80 mM NaPO_4_, and 0.3 mM hydrogen peroxide. The absorbance was measured at 690 nm, and the results were expressed as optical density per mg of tissue.

### 2.13. Proinflammatory Cytokine Determination

TNF-*α* and IL-6 concentrations in the kidney tissue samples were estimated using commercially available kits from R&D Systems, USA. The principle of assay was sandwich ELISA. Absorbance was taken at 450 nm. The protein level of supernatant was estimated and the TNF-*α* and IL-6 levels were expressed as pg/mg of protein.

### 2.14. Assessment of Nitric Oxide

In the assay, nitrate was converted to nitrite by nitrate reductase and total nitrite was measured using the Griess reaction [31]. Briefly, samples were incubated with nitrate reductase (0.2 U/mL), FAD (5 mM), and NADPH (50 mM) for 20 minutes at 37°C. The reaction was stopped by addition of sodium pyruvate (10 mM) and lactate dehydrogenase (24 mg/mL) for five minutes at 37°C and precipitated with 1.4% ZnSO_4_. Total nitrite reacted with Griess reagent (1% sulphanilamide, 2.5% PO_4_H_3_, 0.1% n-naphthylethylenediamine) for 10 minutes at 37°C and was read using the 540 nm filter in a titrated Biotek ELISA reader.

### 2.15. Preparation of Nuclear and Total Protein Extracts

Frozen kidney tissue from different experimental groups was homogenized in ice-cold RIPA buffer containing 1% protease inhibitor cocktail (Sigma-Aldrich) to get total protein extracts. After being centrifuged at 2500 ×g for 20 min at 4°C, the supernatant was collected and used for analysis of cleaved caspase-3, Bax, Bci-2, and *β*-actin expression. Similarly, nuclear extracts were prepared by using NE-PER nuclear and cytoplasmic extraction kit (Pierce Biotechnology, Rockford, IL, USA) containing 1% protease inhibitor cocktail (Sigma-Aldrich) according to the manufacturer's protocol and used for analysis of NF-*κ*B (p65) protein expression as well as NF-*κ*B-DNA binding assay. The protein contents were determined by using Lowry method against a standard curve of a selected standard protein solution bovine serum albumin (BSA).

### 2.16. Analysis of NF-*κ*B (p65) Activation by ELISA

NF-*κ*B DNA-binding activity was analyzed using the NF-*κ*B (p65) transcription factor ELISA assay kit Cayman Chemical Company, Ann Arbor, (USA). Briefly, nuclear extracts were incubated in the oligonucleotide-coated wells where the oligonucleotide sequence contains the NF-*κ*B response element consensus-binding site. After washing, samples were incubated by addition of specific primary antibody directed against NF-*κ*B (p65). A secondary antibody conjugated to horseradish peroxidase (HRP) was added to provide a sensitive colorimetric readout at 450 nm.

### 2.17. Western Blot Analysis

Western blot analysis was performed according to Towbin et al. [32]. The proteins were electrophoretically transferred to PVDF membranes, blocked in 5% skim milk in Tris buffer saline (TBS) containing 1% Tween 20 for 2 hr at room temperature and probed with the polyclonal cleaved caspase-3 (sc-22171-R), Bax (sc-6236), Bcl-2 (sc-492), and NF-*κ*B (p65) (sc-398442) and *β*-actin (sc-47778) polyclonal rabbit-anti rat antibodies as solutions in PBS containing 1% Tween 20 on a shaker for 2 hr at room temperature followed by horseradish peroxidase- (HRP-) conjugated secondary antibodies (1 : 3000) for 1 h and visualization with the enhanced chemiluminescence system Santa Cruz Biotechnology (USA). *β*-actin was used as the loading control for total proteins densitometric analysis of immunoblots was performed with the Image J software (NIH).

### 2.18. Statistical Analysis

Values are given as arithmetic means ± standard error of the mean (SEM). Data were statistically analyzed by using one-way analysis of variance (ANOVA) followed by Dennett's test.

## 3. Results

### 3.1. DPPH Free Radical Scavenging Activity

The ethanolic extract of* B. vulgaris* (BVEE) and fractions were tested for their in vitro antioxidant activity using DPPH radical scavenging assay method with ascorbic acid as a reference. The obtained results are presented in Table 1. The radical scavenging activity of the BVEE showed highest activity (90.9%) at concentrations 500 and 1000 *μ*g/mL, respectively, comparing to ascorbic acid.

### 3.2. Effect of BVEE on Kidney Function Tests

The effect of BVEE treatment on the GM-induced nephrotoxicity on urea, uric acid, total protein, and creatinine levels in serum are shown in Table 2. Administration of GM significantly elevated the levels of urea (28.50 ± 1.17 to 137.00 ± 5.87 nM/L, *P* < 0.001), uric acid (1.11 ± 0.14 to 5.36 ± 0.34 mg/dL, *P* < 0.001), total protein (3.82 ± 0.23 to 8.23 ± 0.14, *P* < 0.001), and creatinine (2.12 ± 0.26 to 9.25 ± 0.46 mg/dL, *P* < 0.001), in serum. Treatment of rats with the BVEE (250 and 500 mg/kg p.o) significantly prevented the elevation of urea, uric acid, total protein, and creatinine levels in serum in dose dependent manner.

### 3.3. Effect of BVEE on the Level of MDA in GM-Induced Renal Damage

**Figure 1:**
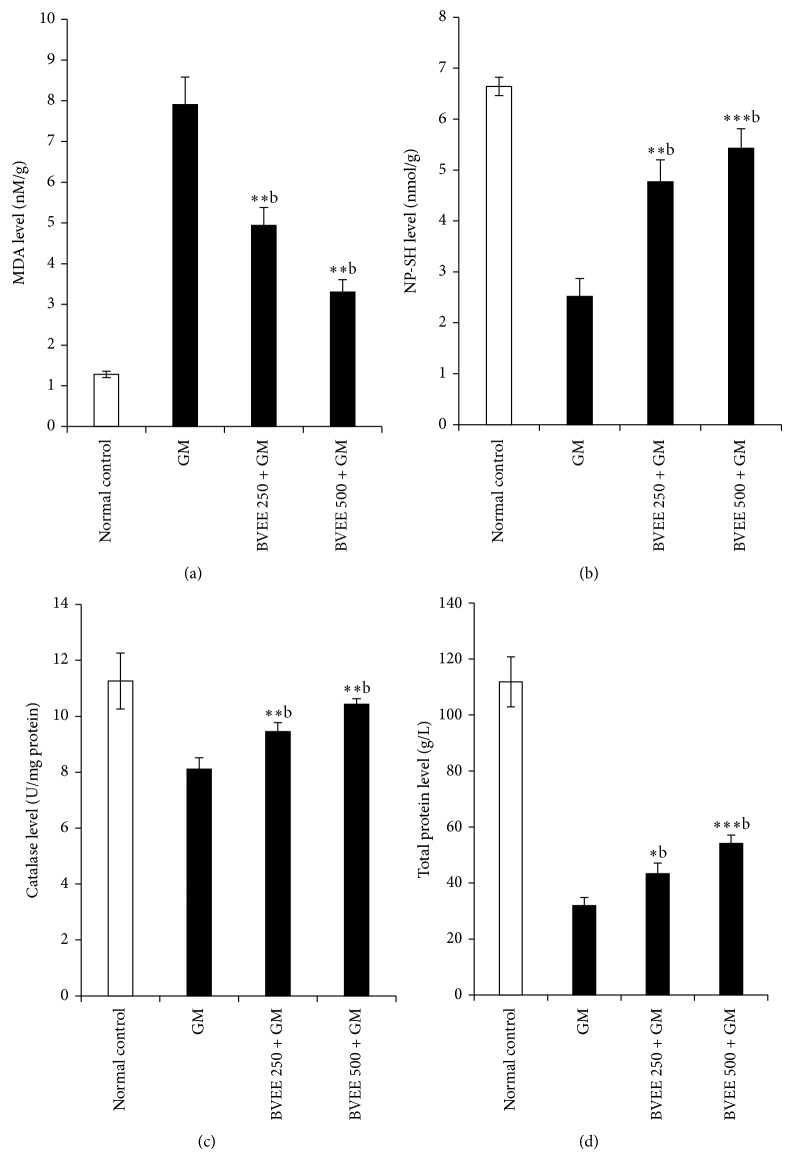
Effect of BVEE (250 and 500 mg/kg; p.o) on gentamicin induced nephrotoxicity on kidney lipid peroxidation: (a) MDA; (b) NP-SH; (c) catalase activity; and (d) total protein. The results are presented as the mean ± SEM. of six animals per group. “∗, ∗∗” ∗∗∗ denotes significant differences from the control group (*P* < 0.05, *P* < 0.01, and *P* < 0.001); “^a,b,c^” denote significant differences from the GM group (*P* < 0.05, *P* < 0.01, and *P* < 0.001).

The extent of lipid peroxidation as measured by formation of (MDA) as depicted in Figure 1(a). There is a sharp increase in (MDA) level in GM treated (Group II) rats (1.28 ± 0.08 to 7.91 ± 0.67) indicative of oxidative stress. The BVEE 250 mg/kg and 500 mg/kg + GM groups III and IV treated rats showed significant dose dependent reduction of MDA level as compared to GM (group II) treated rat. This clearly showed the dose dependent reduction oxidative stress in terms of MDA content by BVEE (250 and 500 mg/kg p.o) 37.54% (4.94 ± 0.44 nM/mg tissue *P* < 0.01), and 58.28% (3.3 ± 0.44 nM/mg tissue), respectively, as compared to GM treated rat in group II (7.91 ± 0.67 nM/mg tissue, *P* < 0.01).

### 3.4. Effect of BVEE on the Level of Reduced NP-SH Content in GM-Induced Renal Damage

GM 85 mg/kg treatment rats to group II showed significant decrease in NP-SH content 37.94% (6.64 ± 0.18 to 2.52 ± 0.35, *P* < 0.001) indicative increase in protein metabolism, whereas there is a significant dose dependent increase in NP-SH content in group III pretreated with BVEE 250 mg/kg + GM 85 mg/kg 71% (6.64 to 4.77 ± 0.43, *P* < 0.01) and group IV pretreated with BVEE 500 mg/kg + GM treated groups 81.71% (6.64 ± 0.18 to 5.43 ± 0.38, *P* < 0.001) in comparison to GM treated group II. This clearly indicates that BVEE 250 and 500 mg/kg have ability to replenish the NP-SH content to normal levels as shown in Figure 1(b).

### 3.5. Effect of BVEE on Catalase Activity in GM-Induced Renal Damage

GM treatment caused 27.97% significant decrease in catalase activity (11.26 ± 0.997 to 8.11 ± 0.040 U/L, *P* < 0.01), in group II rats as compared to normal control kidney tissue (Figure 1(c)). The pretreatment of BVEE at the doses of 250 and 500 mg/kg resulted in a significant increase of catalase level from (8.11 ± 0.040 to 9.45 ± 0.032 U/L and 10.43 ± 0.191, *P* < 0.01) as compared to group II: therefore, BVEE has ability to replenished the catalase level significantly in dose dependent manner which is about 83.92% and 92.62% for BVEE 250 and 500 mg/kg, respectively.

### 3.6. Effect of BVEE on the Level of Total Protein in GM-Induced Renal Damage

As depicted in Figure 1(d), GM treated rats group II showed significant decrease in total protein, 71.46% (from 111.85 ± 8.93 to 31.92 ± 2.89 g/L, *P* < 0.01), content which is indicative of GM-induced kidney injuries caused due to oxidative stress, whereas there is a dose dependent increase in total protein content significantly in group III pretreated with BVEE 250 mg/kg + GM and group IV pretreated with BVEE 500 mg/kg + GM treated groups in comparison with group III which is about 37.35% (43.27 ± 3.85 U/L, *P* < 0.01) and 43.74% (54.15 ± 2.97 U/L, *P* < 0.001). This clearly indicates that BVEE has ability to induce cell proliferation.

### 3.7. Effect of BVEE on Proinflammatory Cytokine (TNF-*α* and IL-6) in GM-Induced Renal Damage

**Figure 2:**
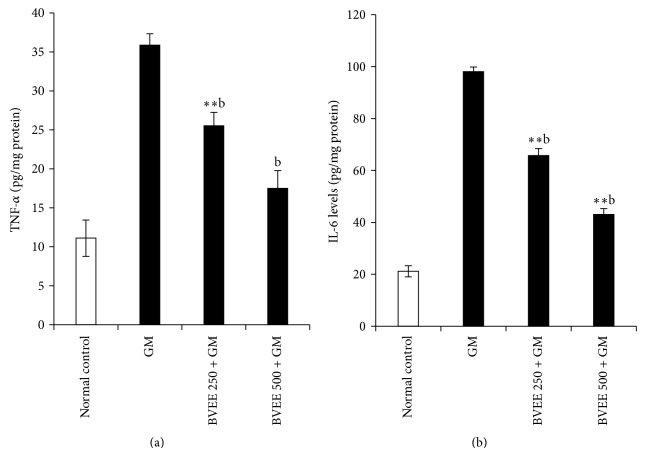
Effect of BVEE on gentamicin-induced changes in proinflammatory cytokines in kidney tissues of rats. (a) Tumor necrosis factor-*α* (TNF-*α*) and (b) interleukin-6 (IL-6). The results are presented as the mean ± SEM of six animals per group. “∗, ∗∗” ∗∗∗ denotes significant differences from the control group (*P* < 0.05, *P* < 0.01, and *P* < 0.001); “^a,b,c^” denote significant differences from the GM group (*P* < 0.05, *P* < 0.01, and *P* < 0.001).

As depicted in Figure 2, GM administration produced a significant 3.2-fold elevation of TNF-*α* (11.11 ± 2.322 to 35.88 ± 1.4, *P* < 0.001) and 4.63-fold elevation of IL-6 (21.13 ± 2.14 to 98 ± 1.77) (Figure 2(a)) levels when compared to group I control rats. BVEE treatment (250 and 500 mg/kg) along with GM significantly (*P* < 0.01) decreased the TNF-*α* levels by 1.4-fold from (35.88 ± 1.4 to 25.54 ± 1.45) when compared with GM control. However, IL-6 levels were significantly (*P* < 0.01) decreased in BVEE treatment at both doses (250 mg/kg and 500 mg/kg) from 98 ± 1.77 to 65.74 ± 2.64, *P* < 0.01, and 98 ± 1.77 to 43.06 ± 2.25, *P* < 0.01. compared to group II GM alone treated rats (Figure 2(b)). These results show that BVEE downregulate the IL-6 level and hence have potent anti-inflammatory activity.

### 3.8. Effect of BVEE on Myeloperoxidase (MPO) Activity and Nitrous Oxide Level in GM-Induced Renal Damage

**Figure 3:**
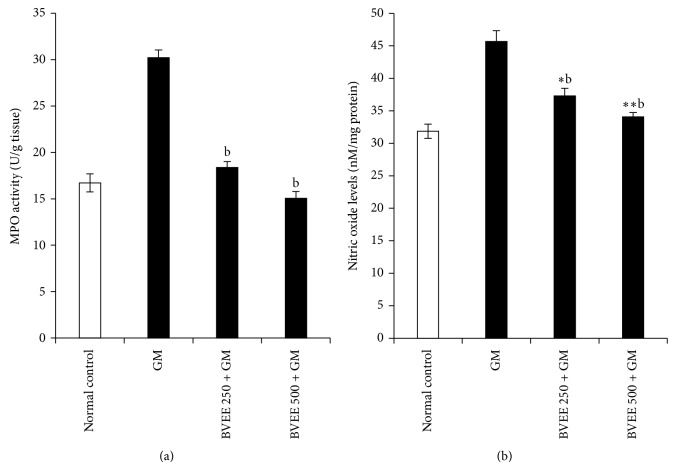
Effect of BVEE on gentamicin-induced changes in inflammatory markers in kidney tissues of rats. (a) Myeloperoxidase (MPO) and (b) nitric oxide (NO). The results are presented as the mean ± SEM of six animals per group. “∗, ∗∗” ∗∗∗ denotes significant differences from the control group (*P* < 0.05, *P* < 0.01, and *P* < 0.001); “^a,b,c^” denote significant differences from the GM group (*P* < 0.05, *P* < 0.01, and *P* < 0.001).

As depicted in Figure 3(a), Myeloperoxidase (MPO) activity was significantly (16.71 ± 0.97 to 30.21 ± 0.83, *P* < 0.01) increased in the kidney tissues of GM alone treated rats when compared to control rats. BVEE decreased the Myeloperoxidase (MPO) activity significantly (18.39 ± 0.63, *P* < 0.01, at 250 mg/kg group III and 15.04 ± 0.74, *P* < 0.01, at 500 mg/kg BVEE group IV) plus GM treated rats in comparison with group II GM treated rats. These results demonstrate the anti-inflammatory potential of BVEE.

To examine the involvement of nitric oxide in the protection against GM-induced renal injury by BVEE; we determined renal nitric oxide levels. As depicted in Figure 3(b) GM treated rats group II showed significant increase in nitric oxide level 43.4% (from 31.85 ± 1.10 to 45.68 ± 1.65 nM/mg protein, *P* < 0.01) content which is indicative of GM-induced kidney injuries caused due to oxidative stress. Whereas, there is a dose dependent decrease in nitric oxide content significantly in group III pretreated with BVEE 250 mg/kg + GM and group IV pretreated with BVEE 500 mg/kg + GM treated groups in comparison with group II which is about 18.38% (37.28 ± 1.19 nM/mg protein, *P* < 0.05) and 25.42% (34.07 ± 0.67 nM/mg protein, *P* < 0.01). This clearly indicates that BVEE inhibits the nitric oxide significantly; hence it has anti-inflammatory properties.

### 3.9. Effect of BVEE on NF-*κ*B in GM-Induced Renal Damage and Apoptosis

**Figure 4:**
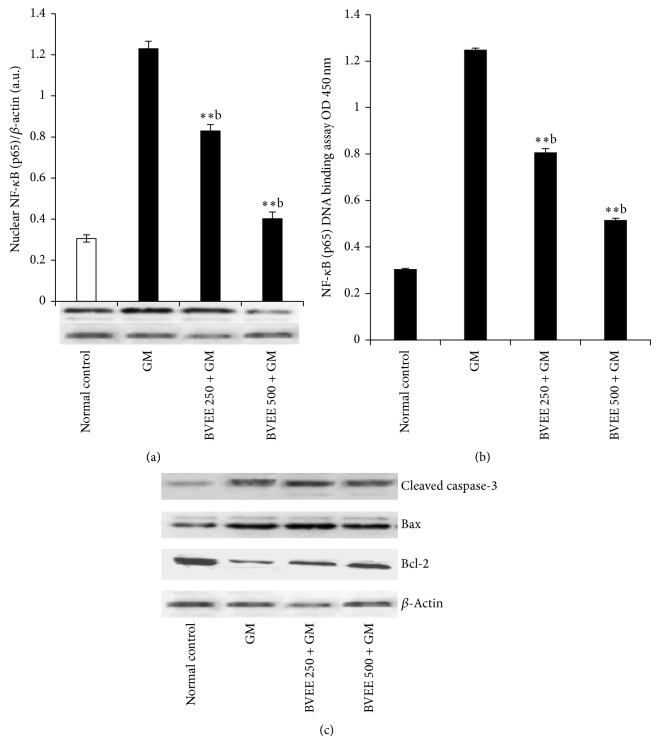
Effect of BVEE on gentamicin-induced changes in inflammatory and apoptotic markers in kidney tissues of rats. (a) Immunoblots analysis of nuclear NF-*κ*B (p65) expression in the kidney tissue of rats treated with BVEE and/or gentamicin. *Β*-Actin expression was used as a loading control. Representative bar diagram showing quantitative relative levels of NF-*κ*B (p65) protein for vehicle, GM, and GM and BVEE treated rats. (b) Nuclear NF-*κ*B (p65) DNA-binding activity determined by using NF-*κ*B (p65) transcription factor ELISA assay kit. (c) Immunoblot analysis of apoptotic marker cleaved caspase-3 and Bax protein and antiapoptotic marker Bcl-2 protein in comparison with *Β*-actin expression was used as a loading control. The results are presented as the mean ± SEM of six animals per group. “∗, ∗∗” ∗∗∗ denotes significant differences from the control group (*P* < 0.05, *P* < 0.01, and *P* < 0.001); “^a,b,c^” denote significant differences from the GM group (*P* < 0.05, *P* < 0.01, and *P* < 0.001).

As illustrated in Figure 4(a), GM significantly increased the nuclear translocation of p65 subunit of NF-*κ*B and NF-*κ*B-DNA binding activity (Figure 4(b)) compared to vehicle control rats. BVEE treatment at 250 and 500 mg/kg significantly reduced both nuclear NF-*κ*B (p65) protein expression and DNA-binding activity when compared to GM alone treated rats. GM administration significantly elevates the renal protein expression of Bax and cleaved caspase 3 activities and significantly (*P* < 0.01) downregulates the Bcl-2 protein expression when compared to vehicle control rats as shown in Figure 4(c).

### 3.10. Histopathological Assessment

**Figure 5:**
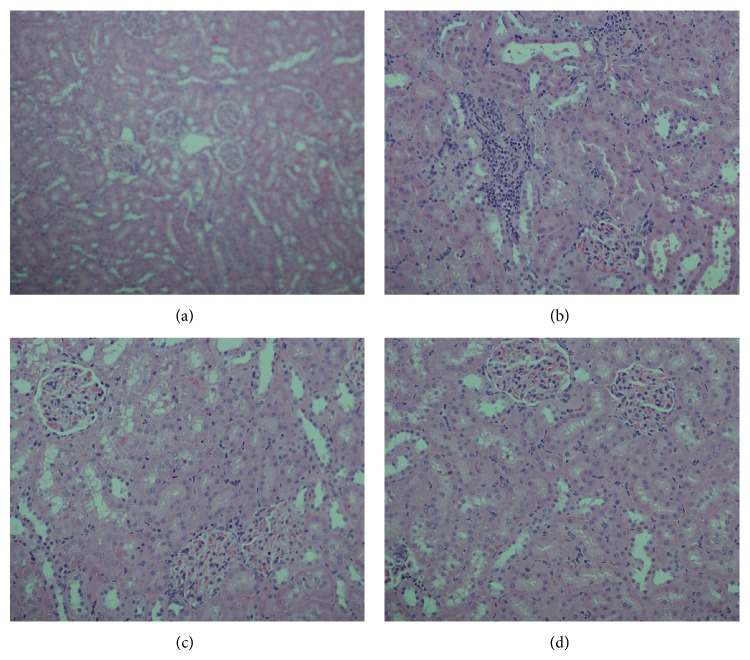
Representative photomicrographs of kidney sections from rats treated with BVEE and gentamicin (H&E staining). Light microscopic examination (×20 objective lens) of kidney tissue. (a) Normal kidney section; (b) effect of gentamicin on kidney tissue showing tubular degeneration, massive necrosis, and focal interstitial nephritis made up of lymphocytes and plasma cells, desquamation of renal tubule epithelium, and vacuolization; (c) effect of BVEE 250 mg/kg, pretreatment showing absence of interstitial nephritis and improvement degree of renal tubule changes; (d) effect of BVEE 500 mg/kg, pretreatment showing almost normal tubules, interstitial, and glomeruli.

Treatment of rats with GM showed a focal interstitial nephritis made up of lymphocytes and plasma cells with desquamation of renal tubules epithelium and vacuolization. Simultaneous treatment of GM-induced nephrotoxic rats with BVEE high dose maintained almost normal tubules, interstitial nephritis, and glomeruli (Figure 5).

## 4. Discussion

Gentamicin is an aminoglycoside antibiotic used against the sepsis in human, but it causes acute renal failure in 10–15% of the patients [33], while more than 30% of the patients showed the signs of nephrotoxicity which have received the gentamicin for more than 7 days [34]. However, due to the selective accumulation of GM in renal cortex [35] leading to nephrotoxicity, its use has been restricted. GM-induced nephrotoxicity causes significant increase in the serum level of kidney function markers such as creatinine, urea, and uric acid as compared to respective control indicating renal dysfunction [36–39]. Administration of BVEE along with gentamicin caused significant decrease in the concentration of creatinine, Urea, and uric acid, while a significant increase in total protein of serum suggested the protective effects of BVEE. Similar protective studies of different extracts against gentamicin have been reported previously [36, 40]. Treatment of GM causes significant increase in the serum level of kidney function markers such as creatinine, Urea, and uric acid as compared to respective control indicating renal dysfunction. As the serum creatinine, urea and uric acid are the ultimate metabolites of purine which may alter the glomerular filtration rate and lead to enhancing their levels in serum and associated with renal damage [41]. Several report suggesting a possible role of oxidative stress in gentamicin-induced kidney dysfunction [36, 42]. Depletion in the CAT, TP, and NP-SH after GM exposure has been reported in other studies suggesting that oxidative stress is one of the causes of renal injuries induced with GM toxicity in rats [43]. Several studies indicate that antioxidant enzymes, including NP-SH and CAT, are inactivated by ROS, including superoxide anion radical and hydrogen peroxide [44, 45].

MDA is an end product of LPO. In this study, an increased level of MDA concentration which is an important pathogenic factor(s) that injures biomembranes was observed [46]. Treatment of BVEE to rats in combination with gentamicin reversed all these alterations suggesting that BVEE rendered its antioxidant properties by preventing or scavenging the ROS induced with GM toxicity. Antioxidants like Vitamin C and E were commonly used to reduce the GM-induced nephrotoxicity [47, 48]. In recent years efforts are focused on the development of antioxidants from natural sources including, herbs, vegetables, and fruits which are able to minimize the toxic effects of GM on kidney [13, 49]. Our results are in accord with other studies where antioxidant effects of other plants have been reported against gentamicin induced renal damage [50, 51]. There are several reports which suggest that beet root extract has beet pigment known as betalains. The betalains found in beetroot were vulgaxanthin I, vulgaxanthin II, indicaxanthin, betanin, prebetanin, isobetanin, and neobetanin. Also cyclodopa glucoside,* N*-formylcyclodopa glucoside, glucoside of dihydroxyindole carboxylic acid, betalamic acid, l-tryptophan,* p*-coumaric acid, ferulic acid, and traces of unidentified flavonoids were detected [10, 52], in addition to the pigments, the root contains oxalic acid, and ascorbic acid. Our findings in* in vitro* antioxidant assay (DPPH) showed a potent antioxidant activity, which further supports its radical scavenging activity. The observed antioxidant and renal protective effect is considered to be related to the phytoconstituents of beetroot such as betalains, terpenoids, sugars, phenolics, and ascorbic acid [10, 53–55]. All these constituents are known to reduce lipid peroxidation and prevent necrosis which protected the kidney tissue against the oxidative damage and nephrotoxic effect caused by GM treatment [1, 56, 57].

Previous studies have reported that renal damage as a result of GM-induced tubular necrosis stimulates inflammatory events at the site of injury leading to enhance the migration of monocytes and macrophages to the site of tissue damage [16].

Myeloperoxidase (MPO) activity in the renal cortex is an index of neutrophil accumulation; therefore, an increase in MPO activity reflects the inflammatory infiltration to the site of tissue injury [58]. In the present study, GM treatment increased the levels of renal MPO activity, indicating neutrophil infiltration. BVEE treatment decreased renal neutrophils infiltration as evidenced by suppression of renal MPO activity and the improvement of histological features. The above findings indicate that BVEE has ability to ameliorate the GM-induced nephrotoxicity through its anti-inflammatory effect. The role played by NO in nephrotoxicity is thought by some as controversial, although some workers have reported that it increased renal injury through its reactions with a superoxide radical and generation of a cytotoxic peroxynitrite [59]. GM treatment induced the nitric oxide level, therefore, increasing the inflammation leading to cellular damage the BVEE treatment significantly attenuate nitric oxide induced oxidative stress leading to reduction in inflammation as well as vasoconstriction of endothelial cells [60]. The activation and nuclear translocation of NF-*κ*B, in response to oxidative stress/nitrosative stress, are the key factors in the renal inflammatory process by regulating the gene expression of cytokines, chemokines, and adhesion molecules [17]. In this study, BVEE (250 and 500 mg/kg) treatment along with GM significantly downregulates the renal nuclear protein expression of NF-*κ*B and NF-*κ*B-DNA binding activities as well as proinflammatory cytokines (TNF-*α* and IL-6) in dose dependent manner as compared to GM alone treated rats. Our findings corroborate those of earlier studies demonstrating that an increase in NF-*κ*B activation is also followed by an increase in the concentration of inflammatory cytokines like TNF-*α* and IL-6 [17, 18]. Beet root extract is able to counteract proinflammatory cascades in peripheral blood mononuclear cells [2].

Apoptosis plays a key role in inflammatory process as well as various renal disease and drug induced nephrotoxicity [61–63]. The prolonged GM treatment may lead to acute renal failure with acute tubular necrosis [21, 64]. Caspase-dependent apoptotic signaling has important role in GM-induced apoptotic renal damage. Caspase-3 can be activated by caspase-9 in the mitochondrial pathway [62]. Bax acts as a proapoptotic protein, whereas Bcl-2 acts as an antiapoptotic protein. The Bcl-2 proteins bind to the outer membrane of mitochondria and block cytochrome c activation [65].

In this contest, oxidative/nitrosative stress seems to play a major role in mitochondrial dysfunction which is an important early event in the intrinsic pathway of apoptosis [66]. GM administration upregulate the protein expression of cleaved caspase 3, Bax and down regulated Bcl-2 expression. This finding clearly shows that GM treatment triggers both apoptosis and inflammation leading necrosis in renal tissues. The BVEE treatment to GM treated rats significantly prevented renal tubular apoptosis/necrosis when compared to GM alone treated rats. Previous studies have shown that NF-*κ*B activation promotes GM-induced apoptosis in rat renal tubular cells [21, 62]. It is guessed that the proapoptotic character of NF-*κ*B might be due to its direct activation of apoptotic proteins such as caspase-3 or downregulation of antiapoptotic proteins such as Bcl-2 [62]. Thus, the result of the present study revealed that BVEE attenuated NF-*κ*B activation; NF-*κ*B DNA binding and renal tubular apoptosis/necrosis associated with GM-induced renal damage. These findings are also in agreement with previously reported literature [11, 18, 61, 66, 67]. Histopathological examination of the GM treated kidney revealed the focal interstitial nephritis made up of lymphocytes and plasma cells with desquamation of renal tubule epithelium and vacuolization. Concomitant use of BVEE with GM ameliorated the renal toxicity induced by GM and resulted in the restoration of histological changes. The potent antioxidant activity of BVEE has been previously reported maybe due to presence of betacyanins including betanin and betanidin [52, 68].

In conclusion, the present study suggests that BVEE has a renal protective potential. The nephroprotective effect of BVEE against GM-induced renal toxicity may be ascribed to its antioxidant, antiapoptosis, and anti-inflammatory properties. These finding substantiate the use of beetroot extract in Arab traditional medicine for the treatment of renal disorders.

## Figures and Tables

**Table 1:** Free radical-scavenging activity of the BVEE, fractions, and ascorbic acid (DPPH-assay).

Treatment	Radical scavenging activity (%)				
	10 *µ*g/mL	50 *µ*g/mL	100 *µ*g/mL	500 *µ*g/mL	1000 *µ*g/mL
BVEE	25.0	27.0	38.4	90.1	90.9
Ascorbic acid (STD)	41.0	86.4	95.5	98.1	98.3

**Table 2:** Effect of BVEE on nephrotoxicity markers in serum.

Treatment	Dose (mg/kg)	Urea (nM/L)	Uric acid (mg/dL)	Protein (g/dL)	Creatinine (mg/dL)
Normal (control)	—	28.50 ± 1.17	1.11 ± 0.14	8.23 ± 0.14	2.12 ± 0.26
Gentamicin	85	137.00 ± 5.87^∗∗∗a^	5.36 ± 0.34^∗∗∗a^	3.82 ± 0.23^∗∗∗a^	9.25 ± 0.46^∗∗∗a^
BVEE + gentamicin	250	120.83 ± 4.40^∗b^	4.19 ± 0.32^∗b^	4.98 ± 0.36^∗b^	7.12 ± 0.59^∗b^
BVEE + gentamicin	500	80.31 ± 4.63^∗∗∗b^	2.77 ± 0.21^∗∗∗b^	6.37 ± 0.50^∗∗b^	4.90 ± 0.33^∗∗∗b^

All values represent mean ± SEM. ^*^ *P* < 0.05, ^**^ *P* < 0.01, ^***^ *P* < 0.001; ^a^ *P* < 0.05, ^b^ *P* < 0.01, ^c^ *P* < 0.001. ANOVA, followed by Dunnett's test.

^∗,∗∗,∗∗∗^As compared with normal group. ^a,b,c^As compared with GM only group.
